# Producing or reproducing reasoning? Socratic dialog is very effective, but only for a few

**DOI:** 10.1371/journal.pone.0173584

**Published:** 2017-03-23

**Authors:** Andrea Paula Goldin, Olivia Pedroncini, Mariano Sigman

**Affiliations:** 1 Laboratorio de Neurociencia, Universidad Torcuato Di Tella, Buenos Aires, Argentina; 2 Consejo Nacional de Investigaciones Científicas y Técnicas (CONICET), Ministry of Science, Buenos Aires, Argentina; University of Westminster, UNITED KINGDOM

## Abstract

Successful communication between a teacher and a student is at the core of pedagogy. A well known example of a pedagogical dialog is ‘Meno’, a socratic lesson of geometry in which a student learns (or ‘discovers’) how to double the area of a given square ‘in essence, a demonstration of Pythagoras’ theorem. In previous studies we found that after engaging in the dialog participants can be divided in two kinds: those who can only apply a rule to solve the problem presented in the dialog and those who can go beyond and generalize that knowledge to solve any square problems. Here we study the effectiveness of this socratic dialog in an experimental and a control high-school classrooms, and we explore the boundaries of what is learnt by testing subjects with a set of 9 problems of varying degrees of difficulty. We found that half of the adolescents did not learn anything from the dialog. The other half not only learned to solve the problem, but could abstract something more: the geometric notion that the diagonal can be used to solve diverse area problems. Conceptual knowledge is critical for achievement in geometry, and it is not clear whether geometric concepts emerge spontaneously on the basis of universal experience with space, or reflect intrinsic properties of the human mind. We show that, for half of the learners, an exampled-based Socratic dialog in lecture form can give rise to formal geometric knowledge that can be applied to new, different problems.

## Introduction

Imagine you want to duplicate the area of a 2 × 2 square. How long should the side of that new square be? Can you accurately draw that new square?

This question was first posed almost 25 centuries ago by Socrates to an illiterate slave [1], culminating in a dialog that constitutes the first educational method written in the history of humanity: ‘Meno’. This dialog [2] and the methodology introduced by it continue to be used nowadays [3, 4]. For the last few years we have been applying a contemporary version of the dialog and asking its central question to 21st century people in our Lab. We demonstrated the universality of this classical model, confirming that a contemporary version of the Socratic dialog shows a remarkably similar trajectory, reflecting the same pattern of errors and correct responses [5, 6].

In a Socratic dialog, the role of the teacher is to encourage the learner to explore a given topic by asking thought-inducing questions and creating contradictions by posing counter examples, while avoiding giving her own answer. Though teacher and learner play active parts in the construction of the dialog, it is the teacher who guides the dialog to the solution [7].

The ‘Meno’ dialog consists of fifty questions posed by Socrates (or the experimenter) and answered by the participant. Basic geometrical principles that underlay Pythagoras’ theorem are explored through these questions, which are driven by a main question: *how to double the area of a given square* (for a complete version of the experimental dialog and protocol used in the lab, see [5]). Briefly, both the original and the experimental dialog start with a draw of a 2 × 2 feet square followed by the simple question ‘Do you know this is a square?’. After a few questions and answers, it is agreed that if a square were to duplicate the area of that 2 × 2 square, the resulting square’s area should be eight. At that moment the participant is asked for the first time about the precise length of a side of the new double-square. Throughout the dialog participants can make mathematical mistakes (e.g., the first time Socrates asks the length of the side of the double square, him and the slave elaborate on the fact that duplicating the side of a square should quadruply its area), and go back and forth between the fifty questions to finally (correctly) answer that the side of the resulting square would be the diagonal of the original square.

**Fig 1:**
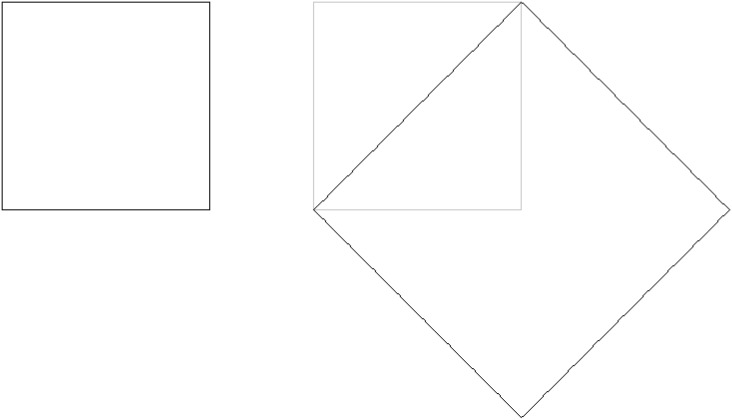
The ‘Meno procedure’. The ‘diagonal argument’ allows to draw a square whose area exactly doubles (right, black) the 2 × 2 original square (left).

The learning experience ends with all participants showing that the area of a square can be doubled using its diagonal as the side of the new square (Fig 1). The last three questions of the dialog (48 to 50) restate this point in different ways and reinforce the correct answer.

The first system of formal geometry that students learn is mostly based on the formal axiomatic geometry created by Euclid 2300 years ago. The principles of this system agree best with human intuition [8]. Socrates argued that Euclidean geometry comes naturally to the human mind. And even today, secondary school geometry assumes that students think on a formal deductive level. However, this is often not the case [9]. Even after the geometrical reasoning underneath a problem is explained, students tend to manipulate it by rote and operate on it by replicating processes that they do not understand.

Geometry is the part of mathematics for which successful problem solving relies on the conceptual understanding of properties of a variety of objects including squares, triangles, rectangles, etc. This conceptual understanding takes time to develop [10]. Two fundamental properties of Euclidean geometry, length and angle, provide good examples of how this knowledge is acquired. Around 4 years of age, children understand the relations between the shapes of objects only with respect to their length. It is only later in development that children start to understand different object representations on the basis of their angles [8]. The mere course of development is not enough to acquire an understanding on basic Euclidean geometry. Formal education plays a central role in driving the attention of students to specific geometric properties.

Learning and transfer are cornerstones of education. Learning is the process by which knowledge is increased or modified, while transfer is the process of applying knowledge to novel situations beyond those in which it was acquired. Making connections between procedures and their conceptual basis increases the understanding and enables students to transfer their knowledge to similar problems [11–13]. The importance of learning is illustrated by a crucial moment of the dialog: Socrates, the teacher, points to the student the diagonal of the original square to make him reflect on how this may be useful in the construction of the new square. This step is referred as the ‘diagonal argument’ –which is the essence of the final and main line of reasoning. However, for an educational process to be successful, it should promote transfer. The main goal of our version of the dialog is to study how the conceptual knowledge acquired in the original dialog is transferred to novel problems.

It is important to highlight the distinction between different kinds of mathematical knowledge that, more often than not, work together in helping us solve geometry problems. One is the *procedural performance* or competence a student has in certain domain; it refers to the knowledge of operators and symbolic representations of mathematics and the use of algorithms and rules to reach certain goals [13]. The other kind is the knowledge or *understanding of concepts*; it refers to the principles and relationships that guide the successful solving of a problem, and its acquisition calls for meaningful and abstract thinking processes.

If students do not have sufficient procedural fluency, they may experience difficulties in solving geometry problems and approving exams, which regularly test procedural performance. Though students are usually more successful in procedural rather than conceptual tests [12], both kinds of knowledge are critical for academic achievement in geometry and there is still some debate on whether geometric concepts emerge spontaneously on the basis of a universal experience with space or reflect intrinsic properties of the human mind [8, 14, 15]. In our paradigm, we are interested on the acquisition of conceptual knowledge more so than evaluating the proficiency in applying previously acquired procedural knowledge. In consequence, our central claim is that participants who have followed the dialog successfully by responding heuristically (i.e., based on prior knowledge and beliefs) are not undergoing the reasoning processes which might result in the concrete learning and understanding that might allow the transfer of the knowledge acquired to new geometric problems. To transfer what happens to one square to ‘all squares’ involves making explicit the reasons for the truth of the assertion that all squares are equal, by means of operations or transformations on a square that was not presented in the experimental dialog but which has all the characteristics representative of its class. In solving this dialog, do students learn geometrical rules more abstract than the factual content of it? Furthermore, with the double-square problem, can we identify something that, once learnt, will make easier to learn more general knowledge? The ultimate question is whether a teacher could use the content taught in the dialog as a vehicle for deliberately teaching transferable general concepts and skills [16].

In our previous work we tested this claim empirically. After following the dialog for more than 20 minutes, we gave participants a ‘different’ square and asked them the same question that led the dialog: to find the side of a square whose area was the double of that of the new square. Puzzlingly, we found that half of the teenagers and almost one third of the adults failed to answer the question [5, 17]. This allowed us to divide participants in two kinds: those who understood that ‘the diagonal argument’ is a rule that can be applied to all squares (we call them Transfer Group, as they built a conceptual knowledge), and those that were incapable of generalizing that knowledge even though they had succeeded the procedural test during the dialog (Non-transfer Group).

In our current project we aim at providing further evidence in support of this finding and to extend them to a more pragmatically pertinent learning situation: the classroom. A significant concern regarding an educational method that has proven to only be effective for a fraction of participants –those who were close enough to benefit from that learning, is whether it would work in a classroom environment, in a ‘real life’ setup. We investigated this idea by giving a ‘Meno-Socratic-lesson’ to an experimental and a control classes, and tested whether the one-to-one dialog triggers similar results when it is used with a group of students.

**Fig 2:**
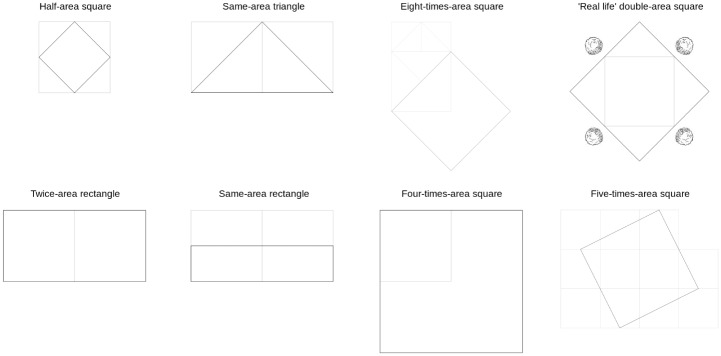
The eight pictorial problems and one (of their many) solutions. The original square for each draw is light gray, while the solution is black. Top row: Diagonal-related problems (DR); Bottom row: Diagonal-non-related problems (DnR).

Although in our previous work a wide part of the students could not use the information acquired during the dialog to solve an almost identical problem, after following the dialog the majority of individuals could transfer the original double-square problem to the new double-square problem. But this raises an important question: what did they learn? Several possibilities come to the fore: they learnt to just double a square, they learnt to reason through any geometrical problem involving the use of the diagonal, or they learnt to duplicate areas. In our current project we seek to clarify this issue by exploring the boundaries of what was learnt. To that aim, in addition to asking them to solve the central problem of the dialog, we tested all participants with a set of eight problems of varying difficulty (Fig 2); four of them that could be solved using the diagonal argument, and four that could not.

Our core prediction was that those participants that could learn how to double ‘any’ square have learnt something more profound than just how to double a square: they have acquired a novel form to establish relations between object representations. They learnt to apply the geometrical reasoning that sustains these relations, and, more specifically, to use the diagonal to solve area problems. In this project we aimed at providing further empirical support for this claim about the nature of geometrical learning by delimiting the boundaries of the transfer process elicited by the dialog within a classroom setting.

## Materials and methods

### Participants

This experiment was conducted at a summer science camp for teenagers [18] in 2015. 81 teenagers (14-to-18 years old) were blindly assigned to one of two camps that took place one week apart in Patagonia (groups were matched by gender and age). One of the camps was randomly defined to become the Experimental class (n = 41, from 14 to 18 years old: n = 3, 11, 14, 12, and 1, respectively; 20 males), while the other one was the Control (n = 40, from 14 to 17 years old: n = 7, 8, 13, and 12, respectively; 19 males).

All the data were collected and treated anonymously; a random identification number was assigned to each participant. This experiment is framed under a protocol authorized by the institutional Ethical Committee of the Centro de Educación Médica e Investigaciones Clínicas, Consejo Nacional de Investigaciones Científicas y Técnicas, Buenos Aires, Argentina. Additionally, for participants under the age of 18, legal guardians gave written consent for their participation in all camp activities including their participation in research studies. The consent was signed in front of a competent legal authority of Argentina in order to assure that it was not adulterated. The research that provided the data for this manuscript was part of the formal curriculum of the camp. The researchers that performed the experiment and collected the data were formal teachers of the camp and legal guardians knew the scientists were in charge of the activities. Finally, all the procedures were approved by the NGO Board of directors. Before the start of the experiment, teenagers were notified that the activity would be an experiment to be used for research purposes, that they could abandon it at any moment, and all research procedures were explained. All teenagers gave oral consent and completed the experiment.

Nine trained subjects were excluded from the analysis because they could not answer the last question of the dialog, and we are not certain about the amount of attention they had paid to the lecture.

### Training and testing procedures

In each class, all participants sat side by side in a classroom. At all times, participants were not allowed to speak except to ask clarification questions out to the class. The Experimental class underwent a 40-minutes lesson (see below) while the Controls did a quiet activity for approximately the same amount of time.

A researcher gave a lecture to the Experimental class in a format that resembled a typical school lesson. It was adapted from the original dialog, as closely as possible, but some modifications were introduced to accommodate to the group lecture setting. For instance, as the experimenter did not know the individual answers of each participant, she would say: ‘Many people think that the side should be 4. Let’s see what happens if it is 4’. Teenagers were provided with pen and paper, and required to answer in writing every time the experimenter posed a question. Questions were previously established as a subsample of those included in the original dialog and there were 23 of them.

After the Experimental lesson or the Control activity, all participants received nine questions. Each of the questions was presented separately and all questions were assembled in a booklet. Participants were instructed to answer one question at a time and as accurately as possible both in graphical and written form. If they could not answer a question, they were asked to elaborate on their difficulties for doing so. A 3-minutes maximum time was allotted for each answer in order to control the ‘thinking time’ variable (based on prior piloting). Every 3 minutes the experimenter told the participants to continue to the following question. A warning was given 30 seconds before the 3 minutes mark. If participants finished before the 3 minutes had elapsed, they had to wait and could not look at the following question nor go back to a previous one. Research assistants controlled that participants followed these rules.

The following nine problems were posed in order. Given an original 2 × 2 square: 1) draw a square whose area exactly doubles the original square; 2) draw a square whose area is exactly half of the original square; 3) draw a triangle whose area is exactly the same as the original square; 4) draw a square whose area is exactly eight times the original square; 5) draw a rectangle whose area is exactly twice the original square; 6) draw a rectangle whose area is exactly the same as the original square; 7) draw a more concrete version of problem *1* [‘The pool problem’: A ‘pool’ was represented as a square with four ‘trees’ located on the outside, each tree next to each vertex of the pool (see fourth picture of Fig 2); participants were told that the owner of the pool wanted to duplicate its area without throwing down any tree, asked whether that was possible and, if so, how could it be done.]; 8) draw a square whose area is exactly four times the original square; and 9) draw a square whose area is exactly five times the original square. One of the possible solutions for each problem is depicted in Fig 1(problem *1*) and Fig 2(problems *2-9*).

### Data analysis

The answers provided by the participants were coded on a binary basis (1: geometrically correct; 0: geometrically incorrect or not answered). A tenth question was excluded from the analyses because more than 86% of the participants reported not understanding it. A performance measure was calculated as the percentage of participants who answered correctly each of the 9 questions included. An answer was considered correct when participants had provided an explanation (in graphical or written form) of why they were certain that the figure they draw was the exact size as the figure requested. In 5.8% of responses, participants wrote square roots without explaining their meaning. Those answers were excluded from the analysis given that it was impossible to establish if those participants understood the reasoning behind the problem. 62 participants, 76%, did not use square roots in any of the problems. In the results, we report the two-tailed P-values for Fisher’s exact test.

## Results

Results showed that the experimental group had followed the lecture (97.4% of all the questions posed by the experimenter were answered). All experimental participants answered incorrectly the first and the second time the double square question was posed during the dialog, proving that they did not know beforehand the argument that the dialog was attempting to teach.

To verify whether the Dialog-turned-lecture elicited the same near-transfer effect than the original experimental dialog, we first compared the responses for question 1 of both the Experimental and the Control groups (see Fig 1). While half of the participants in the Experimental group correctly marked the diagonal of the new square (57%), none of the participants in the Control group did so. Furthermore, the proportion of incorrect answers of the Experimental group in this ecological situation was not different from our previous findings (43%) [5]. Hence, for the following analyses we divided the experimental participants in two groups according to their responses to question 1: *Trained-Transfer* and *Trained-Non-Transfer*.

In order to investigate the boundaries of the double-square transfer process, the 8 problems given to participants were intermingled conforming two different groups (Fig 2). The first group (*Diagonal-related problems*, DR) was to verify whether the learning elicited by the lecture extends to similar forms of geometric reasoning involving diagonal arguments (problems *2, 3, 4, 7*). We predicted that correct answers involving the diagonal reasoning combined with translations and rotations would increase after the lecture only for those participants that understood the conceptual geometric notions that underlay the dialog.

The second group (*Diagonal-non-related*, DnR) included problems that do not require operations with the diagonal (problems *5, 6, 8, 9*) and allowed us to test whether the transfer extended to other area arguments.

**Fig 3:**
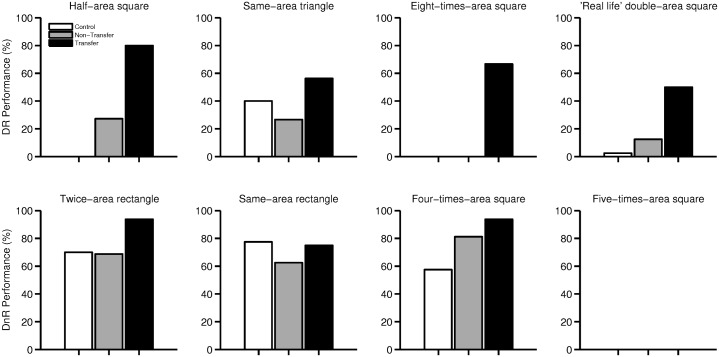
Participants that answered correctly the eight questions to test far transfer. Black bars show participants that, after the lecture, had transferred the original problem to a new square (Fig 1). White or grey bars show participants that could not do that transfer (from the Control and the Experimental classes, respectively). No Control participant could transfer the original problem. Top row: Diagonal-related problems (DR); Bottom row: Diagonal-non-related problems (DnR).

To test the further transfer to other geometrical problems of the learning elicited by the lecture, we compared the responses of the Experimental and the Control groups in the eight problems. As previously mentioned, the response to question *1* (that determines near transfer) was used as a proxy for transference. Fig 3 shows the performance achieved in each of the two groups of problems.

We first compared the performance of each experimental group for both types of questions and found a significant difference for DR questions (*χ*^2^(2, *N* = 265) = 64.98, *p* = 0), but no difference for DnR questions (*χ*^2^(2, *N* = 277) = 4.60, *p* = .10). Follow-up analyses showed no difference between the Control and the Trained-Non-Transfer groups (*χ*^2^(1, *N* = 26) = 0.98, *p* = .32), and a significant difference between the Trained-Transfer group and, both, the Control and the Trained-Non-Transfer groups (*χ*^2^(1, *N* = 56) = 59.55, *p* = 0, and *χ*^2^(1, *N* = 48) = 25.44, *p* = 0, respectively).

Furthermore, the percentage of correct answers for DR is smaller than that of DnR problems, indicating that the former are harder (although nobody could properly answer the last of the DnR questions), Table 1.

**Table 1:** 

Percentage of correct responses			*χ*^2^	*p*
	DR(*a*)	DnR(*b*)		
*ControlGroup*	11.41	52.90	59.56^+^	0.00
*Trained*–*Non*–*Transfer*	16.67	55.74	18.68^+^	0.00
*Trained*–*Transfer*	62.90	68.85	0.48^+^	0.49

^a^Diagonal-related problems.

^b^Diagonal-non-related problems.

^+^ *df* = 1.

Considering the DnR problems independently, the only one that showed a significant effect for Trained over Control participants is the one that required to quadruply the area of a square (*χ*^2^(1, *N* = 72) = 7.74, *p* = .0054).

When considering the DR problems independently, the only one that showed no difference between groups is the one that requires to build a triangle of the same area as a square (*χ*^2^(1, *N* = 71) = .0062, *p* = .94). We included it as part of the DR problems because an easy way to solve it would be to ‘cut’ in half the square using the diagonal, and to put both diagonals next to each other (Fig 2). Interestingly, half of the 44% of the Trained participants that answered this question correctly, didn’t use the diagonal approach: they answered with the learnt-at-school formula *base* × *height*.

## Discussion

We have shown that the capacity of the Socratic dialog to convey geometric knowledge is not changed when the dialog is used in a more typical educational context of one teacher providing it to a large group of students. As in the laboratory situation, some people really learn to solve the problem, while nearly half do not. Our results also show that what is learnt in the dialog can be transferred to other geometrical problems as long as both types of tasks satisfy two properties: a) they share some reasoning aspects (i.e. involve the diagonal reasoning combined with translations and rotations), and b) they are not extremely difficult (i.e. can be solved within the short time allowed). These results indicate that the dialog leads to a transfer process that goes further than doubling squares’ areas: the knowledge acquired can be used to solve other problems that require the ‘diagonal reasoning’. Our results also provide evidence that the one-shot near-transfer question (problem *1*) is a good proxy for assessing proper learning of the ‘diagonal argument’ and far transfer.

The comparison of the performance of participants that had not been exposed to the lecture with the performance of those ones that pass through the class without transferring, allowed us to formulate a critical prediction: if the Socratic dialog can be used residually, the Non-Transferrers should have benefited from the dialog for the diagonal related problems. Participants that did not experience the lecture should in average have better previous knowledge than the non-Transferrers, because of a random selection effect (about 40% of them would have benefited from the dialog). Hence, we should see an interaction between the category of problems posed (related or not related to the diagonal) and having been exposed to the dialog; where the overall trend should be that the Non-Transferrers showed better performance for diagonal related problems, but worse performance for the other geometric problems. However, this is not what we found: participants that were not exposed to the dialog and those that did experience the lecture but failed to transfer knowledge were indistinguishable from each other. Moreover, the 9 excluded subjects that could not answer the last question of the dialog were indistinguishable from either those that did not experience the dialog or those that failed to transfer knowledge after experiencing the dialog (*p* > .66 in all cases).

Of the problems not related to the diagonal, the only one that showed a positive effect of having experienced the dialog was to find a square four times the area of the original square (third comparison of the second row of Fig 3). This is not so puzzling because, though there is no need to use a diagonal reasoning to properly answer that question, this drawing appeared during the dialog and was casually drawn twice by the experimenter. However, it suggests that the reasoning elicited throughout the dialog leaves a trace that persists after it is finished. In this same sense, it is surprising that after following the dialog, some participants that failed to duplicate the area of a square could solve further problems, such as the half of the area, or a real-life problem (Gray bars of the first and fourth comparisons of Fig 3, respectively). There could be multiple explanations that account for this effect. We think that a likely explanation is that the time elapsed between the end of the dialog and the answering of these problems might have helped these participants, allowing them to put together the knowledge acquired through the dialog.

The participants that transferred not only generated a different understanding of concepts, but also used that knowledge more effectively. Those that could transfer procedural knowledge to conceptual knowledge (those who extracted the diagonal argument) were able to modify their conceptual knowledge and applied the new concept to other geometrical figures [11]. In previous studies we found enhanced frontal lobe activity in those that failed to transfer compared to those subjects that could transfer [17], suggesting that they might have acquired new knowledge, even though they could not use it to solve the near-transfer question. We speculate that the participants that profit the most from the dialog are those that already had the proper background [5] to incorporate the new knowledge into their mental schemas [19]. Our findings show that participants that could not transfer to another square did not either transfer to any of the diagonal related problems; based on this finding, we can only speculate that the higher brain activity that we previously found in the Non-Transferrers might be reflecting the generation of new knowledge schemas.

High school students are usually not very good at reasoning flexibly about mathematics; upon representation of a problem, rather than drawing inferences about it, they resort to applying previously memorised (sometimes incorrect) procedures. Chazan [20] reviews cases where high school students do not see the generalizable aspects of generic examples in geometric tasks. This observations are relevant for the interpretation of our results, he shows that students are often not aware of the distinctions between empirical and deductive proofs. When students do not understand the abstraction principle that underlies deductive proofs, they fail to realise that a specific deductive proof for one triangle applies to ‘all’ triangles.

The importance of pictorial representations in geometry is emphasized in class and in textbooks, where most geometric concepts are accompanied by diagrams, and students are encouraged to use and draw diagrams when learning about and solving geometry problems [21]. As they are pertinent for education, we explored different pictorial representations in which it was possible to use the ‘diagonal reasoning’. An obvious limitation of this study is that all the geometrical figures we used correspond only to a special class of area problems; it would be interesting to explore in further work whether this newly acquired knowledge helps students acquire other kinds of geometric knowledge more rapidly, or use it more flexibly, to solve problems that refer to other types of objects.

Symbolic arithmetic is a cultural invention [22] and the school-taught geometry domain might also depend on appropriate mathematical language. The Socratic dialog relies on a verbal language-dependent representation of geometry; yet disguised by the absence of formulas. A likely scenario we can speculate upon is that, when geometrical concepts are integrated with language-based representations of numbers, they might help build the cognitive basis needed for transfer to other diagonal related problems.

## Conclusion

In sum, our findings suggest that the Socratic dialog is a very good method of teaching that allows the learner to deeply and further understand underlying geometric principles and to use them in other contexts. However, this only happens for half of the learners, those participants that were prepared to learn and could made the proper connections between their previously acquired procedural knowledge and their ability to understand geometry notions. Those participants did learn something new that they can use in novel contexts. On the opposite side, another group of participants of the same class could not make use of the same procedures that they had applied a few minutes before to solve an almost identical problem, even after having completely followed the dialog. For this group of students the method is not successful and its success might require the use of different or pedagogical methods for teaching geometry. As such, they constitute a target group for the investigation of novel methods that can allow for better integration of previously acquired procedural and conceptual knowledge to new concepts.

The most important take home message of our findings is that they enlighten the testing process. If an examination had stopped after testing the strict following of the dialog (the end of the original dialog Socrates had), 78% of the participants would have passed it. But if the test is meant to assess proper learning and transfer (i.e. that students can use the learnt procedure to solve new problems), then it has to go further. A short test of only one question (almost the same problem) posed immediately after the lecture could show the teacher which students need further attention.

## Supporting information

S1 File Raw data. All the responses and demographic information of participants.
(XLS)
